# Lung ultrasound has limited diagnostic value in rare cystic lung diseases: a cross-sectional study

**DOI:** 10.1080/20018525.2017.1330111

**Published:** 2017-06-07

**Authors:** Jesper Rømhild Davidsen, Elisabeth Bendstrup, Daniel P. Henriksen, Ole Graumann, Christian B. Laursen

**Affiliations:** ^a^ Department of Respiratory Medicine, Odense University Hospital, Odense C, Denmark; ^b^ Research Unit of Respiratory Medicine, Clinical Institute, University of Southern Denmark, Odense C, Denmark; ^c^ South Danish Center for Interstitial Lung Diseases (SCILS), Odense University Hospital, Odense C; ^d^ Department of Respiratory Medicine and Allergology, Aarhus University Hospital, Aarhus, Denmark; ^e^ Department of Radiology, Odense University Hospital, Odense C, Denmark; ^f^ Center of Clinical Ultrasound (CECLUS), Aarhus University, Aarhus N, Denmark; ^g^ Center for Thoracic Oncology, Odense University Hospital, Odense C, Denmark

**Keywords:** DLPD, HRCT, lung ultrasound, rare cystic lung diseases

## Abstract

**Background**: Lung ultrasound (LUS) used to identify interstitial syndrome (IS) and pleural thickening related to diffuse parenchymal lung disease (DPLD) has shown significant correlations with ground glass opacity (GGO) on high-resolution computed tomography (HRCT). However, the applicability of LUS in patients with DPLD subtypes as rare cystic lung diseases has not previously been investigated. This study aimed to observe if distinctive LUS findings could be found in patients with lymphangioleiomyomatosis (LAM), pulmonary Langerhans cell histiocytosis (PLCH), and Birt-Hogg-Dubé syndrome (BHDS).

**Methods**: This single centre case-based cross-sectional study of patients diagnosed with LAM, PCLH and BHDS was conducted at a Danish DPLD specialist centre. Patients underwent clinical examination including LUS. LUS findings were compared to findings scored according to a modified Belmaati score on HRCT and reviewed in consensus between two pulmonologists and one radiologist.

**Results**: Twelve patients with HRCT proven cystic lung disease were included, six with LAM, three with PLCH, two with BHDS, and one with uncharacteristic cystic lung disease. The mean age was 48.7 years (SD ± 15.8). In general all had normal LUS findings. IS could not be found in any patients despite GGO presentation on HRCT among 75% of the patients with a Belmaati in the highest category of 0.76–1.00. Pleural thickening on LUS was present in three patients, but with inconsistent findings.

**Conclusion**: This study indicates that LUS has limited value as a diagnostic tool in patients with LAM, PLCH, and BHDS as normal LUS findings did not rule out severe cystic lung disease.

## Introduction

Lung ultrasound (LUS) has within the past 10 years excelled as a fast and non-invasive examination modality to diagnose and monitor various conditions in the lungs and pleura.[1,2] With LUS diseases, in which the density of the lung interstitium is diffusely increased, can be identified, by demonstration of multiple B-lines, as the so-called interstitial syndrome (IS).[1,3] IS may represent cardiogenic pulmonary oedema, ARDS, interstitial pneumonias, but also presence of interstitial inflammation and fibrosis in patients with diffuse parenchymal lung disease (DPLD).[1] Accordingly, LUS used as a diagnostic tool to identify IS related to DPLD has shown significant correlations with findings on high-resolution computed tomography (HRCT) in patients with different subtypes of DPLD, e.g. acute eosinophilic pneumonia,[4] sarcoidosis, hypersensitivity pneumonitis, idiopathic pulmonary fibrosis,[5] and patients with pulmonary manifestations secondary to connective tissue diseases.[6–8]

Traditionally, disease progression and overall prognosis in DPLDs is evaluated in conjunction with repetitive HRCTs performed prior to clinical follow-ups.[9] This may not only result in several inconvenient hospital attendances but also an increased lifetime risk of cancer development due to a cumulated radiation dose exposure.[10,11] In contrast LUS excels by being a radiation-free procedure that can be performed relatively rapidly in relation to clinical attendance with minimal discomfort, but also a procedure which is easy to assimilate.[12,13] Consequently, LUS may be preferable to diagnose and monitor DPLDs in some settings compared to HRCT.

Lymphangioleiomyomatosis (LAM),[14–16] pulmonary Langerhans cell histiocytosis (PLCH),[16] and Birt-Hogg-Dubé syndrome (BHDS) [17] are rare DPLDs. As in other DPLDs, a common feature of LAM, PLCH, and BHDS is predomination of thin-walled lung cysts causing an overall decreased lung density.[18,19] As the pathoanatomical appearance of these rare cystic lung diseases significantly differs from other DPLDs the presence of B-lines and IS as described in other DPLDs may thus not necessarily be characteristic findings in the rare cystic lung diseases. So far no studies have reported systematic use of LUS in LAM, PLCH, and BHDS. Establishing knowledge on potential characteristic LUS findings in these cystic lung diseases or lack thereof is of significance in order to select the most optimal diagnostic and disease monitoring approach.[4–8]

The aim of this study was to observe whether distinctive LUS findings were present in patients with LAM, PLCH and BHDS.

## Materials and methods

### Study design

This is a single centre case-based cross-sectional study of patients diagnosed with LAM, PLCH, and BHDS.

### Setting

In the Central and North Denmark Region, diagnosis and management of DPLDs is accomplished at the Western Danish Centre for Interstitial Lung Diseases at Aarhus University Hospital. This centre receives unselected patients suspected for DPLD from the primary sector within the local area of Aarhus as well as from all other hospital departments within these regions, and thereby serves as a tertiary specialist centre covering an area of 1.85 million inhabitants.[20] The study took place in 10 March 2015.

### Study cohort

Patients were recruited at the Western Danish Centre for Interstitial Lung Diseases. The diagnoses LAM, PLCH, and BHDS were thus established by either of the following:

- a histological confirmation by bronchoscopy with transbronchial lung biopsies or video assisted surgical lung biopsy; or
- on the basis of a conclusion from the multidisciplinary discussion (MDD) involving pulmonologists, radiologists, pathologists, and rheumatologists, in order to increase the diagnostic precision.[21]

The project was approved by the Regional Ethics Committee of Southern Denmark (Project ID S-20140172), and the Danish Data Protection Agency (14/41600), and was performed according to the Declaration of Helsinki. All participants gave written informed consent.

### Procedure

#### Clinical examination

On a Pneumotrac model 6800 (Vitalograph, Ennis, Ireland), all patients underwent a pulmonary function test in accordance with the European Respiratory Society (ERS) and American Thoracic Society (ATS) standards including FEV1 (forced expiratory volume in 1 sec) and FVC in litres and per cent of predicted (% pred.), and FEV1/FVC ratio.[22] Diffusion capacity of the lung for carbon monoxide (DLCO) was singled out from a previous follow-up less than six months before the examination day. DLCO was measured as a single-breath diffusion lung capacity.

#### LUS assessment

LUS was performed by two experienced physicians (JRD and CBL), both EFSUMB certified in LUS.[23]

Patients were examined in a straight-backed sitting position. The thorax was systematically scanned according to anterior, lateral and posterior chest wall using an adapted approach of the principles described by Volpicelli and Lichtenstein,[1,3] and also used in previous studies from this research group.[2,24] In a vertical and horizontal direction, respectively, the anterior chest wall was outlined from clavicles to diaphragm, and from sternum to anterior axillary line; lateral chest wall from axilla to diaphragm, and from anterior to posterior axillary line; posterior chest wall from margo superior scapula to diaphragm, and from posterior axillary to paravertebral line. The anterior and lateral chest walls were divided into an upper (zones 1 and 4); and lower zone (zones 2 and 3), whereas the posterior chest wall was divided into an upper, middle, and lower zone (zones 5–7) equivalent to a total of seven zones for each hemithorax.[2,24] In each zone the transducer was systematically placed vertically across an intercostal space corresponding to the centre of the specific zone. Supplementary horizontal views of the intercostal space in a given zone were performed in case of abnormal findings using the vertical view. In all 14 scanning zones LUS was performed with a GE Logiq E9 (GE Healthcare, Milwaukee, WI, USA) ultrasound system with a linear transducer using a frequency of 6–15 MHz and with a depth and focus setting of 4 cm and 1.5–2 cm, respectively.

### Selected variables

#### LUS variables

B-lines are defined as vertical reverberation artefacts originating from the pleural line extending uninterrupted to the edge of the screen on the ultrasound machine without fading (previously termed ‘comet-tails’).

The primary outcome variable was dichotomised presence of IS. In accordance with the international evidence based guideline for point-of-care LUS, IS was defined as ≥3 B-lines in >2 anterior or lateral zones on each hemithorax.[1]

Secondary outcome variables were dichotomised presence of the below-mentioned LUS findings obtained from each individual scanning:

- *lung sliding* was defined as the dynamic movement that can be observed when the parietal and visceral pleura slide (pleural line) against each other in synchrony with respiration.
- *pleura thickness* was defined as a thickened pleura or thickened part of the pleural line seen a single frame of the LUS clip.
- *lung consolidation* was defined as visible pathology in the lung parenchyma. Visible lung consolidation was further subdivided based on sonomorphologic characteristics into e.g. pneumonia, embolism, and tumour. Lung consolidations with uncharacteristic sonomorphology were registered as ‘uncharacteristic finding’.[2]

Findings such as visible pathology not related to the visceral pleura or the lung parenchyma itself (e.g. pleural effusion) was registered as ‘other findings’. The LUS records for each patient were independently reviewed by two pulmonologists. The final decision of the specific LUS findings for each patient was achieved when both agreed in the interpretation of the individual LUS finding. In case of disagreement a radiologist made the consensus decision using the same interpretation approach as described above.

#### HRCT

The latest available HRCT prior to examination date for each patient was evaluated and scored according to a modified version of Belmaati et al.,[25,26] which specifically aims to identify dichotomised presence of findings as ground glass opacity (GGO), septal thickening, peribronchial thickening, consolidation, fibrosis, mosaic perfusion, air trapping and bronchiectasis in central and peripheral parts of the right upper, middle and lower lobes, and the left upper and lower lobes corresponding to 10 areas in total.[25,26] Presence/non-presence of a specific finding in each area was scored with 1 and 0 respectively, and calculation of total score for each HRCT-finding was performed by adding only the number of positive findings (i.e. the number with score 1) divided with the number of areas (i.e. 10). As an example patient number 8 had presence of GGO in 8 of 10 areas affecting an area with a score of 8/10 = 0.8. Thus, low and high Belmaati scores represented no or high likelihoods, respectively, of presence of the specific radiological finding. The modified Belmaati score for each patient was obtained by three different physicians (OG, CBL, JRD), who individually assessed each HRCT and subsequently made a score based on consensus principles.

### Statistical analysis

The main outcomes were proportions of specific LUS findings categorised to zones and proportions of specific HRCT findings according to the modified Belmaati score.

Continuous variables as baseline demographic data were expressed, when appropriate, as mean(s) with corresponding standard deviation (±SD), and descriptive categorical data were expressed as proportions (e.g. distribution of the different LUS-findings in which the numerator represents the number of participants with a positive specific LUS-finding and the denominator the number of participants from the specific disease category). All analyses were performed using Stata Release 14.0 (StataCorp, College Station, TX, USA).

## Results

### Baseline findings

In total 12 patients with rare cystic lung diseases accepted participation, of whom six were diagnosed with LAM, three with PLCH, two with BHDS, and one with uncharacteristic cystic lung disease. Six patients had their diagnosis confirmed by biopsy. All LAM patients were females and due to a positive gene test two patients had tuberous sclerosis complex (TSC) associated LAM. The two patients with BHDS were men. The three patients with PLCH were or had been smoking corresponding to 19 pack years (SD ± 15.5), whereas none with BHDS and the majority of LAM patients (83.3%) had ever smoked. Patients were diagnosed on average 61.3 months (SD ± 49.2) prior to the inclusion date, with LAM patients in general having the longest disease duration. Individual baseline characteristics are presented in Table 1.

**Table 1.** Individual baseline characteristics.

	Gender	Age		Extrapulmonary disease			Pack	VEGF-D
Patient	(M/F)	(years)	Disease	manifestation(s)	Positive gene test	Type of biopsy	years	(pg ml^–1^)
1	F	34	Unclass.	–	–	–	18	471
2	F	44	PLCH	–	–	VATS	26	–
3	M	49	BHDS	Renal cysts	+	–	–	–
4	F	38	LAM	TS, skin lipoma, angiomyolipoma	+	–	–	846
5	F	76	LAM	–	–	–	–	–
6	F	61	PLCH	–	–	fTBB	43	–
7	M	46	BHDS	Renal cysts	+	–	–	–
8	F	64	LAM	–	–	VATS	–	> 4000
9	F	36	LAM	–	–	Abdominal biopsy	5	> 4000
10	F	39	LAM	–	–	VATS	–	2512
11	F	71	LAM	TS (genotype), angiomyolipoma	+	–	–	2310
12	M	26	PLCH	–	–	VATS	12	–

Abbreviations: – = none/not performed. M = male. F = female. LAM = lymphangioleiomyomatosis. PLCH = pulmonary Langerhans cell histiocytosis. BHDS = Birt-Hogg-Dubé syndrome. Unclass. = unclassified cystic lung disease. fTBB = forceps transbronchial biopsy. VATS = video-assisted thoracoscopic surgery. VEGF-D = vascular endothelial growth factor D. pg ml^–1^ = pictogram per millilitre.

BHDS patients and the patient with unclassified cystic lung disease had normal ventilation and diffusion parameters (Table 2). Patients with LAM and PLCH exhibited a moderately reduced obstructive ventilation and diffusion capacity.

**Table 2.** Baseline characteristics categorised according to disease entity.

Variable*		LAM	PLCH	BHDS	Unclassified	All
N (%)		6 (50.0)	3 (25.0)	2 (16.7)	1 (8.3)	12 (100.0)
Gender (F:M)		6:0	2:1	0:2	1:0	9:3
Age (years)		54.0 ± 18.3	43.7 ± 17.5	47.5 ± 2.1	34.0 ± 0.0	48.7 ± 15.8
BMI (kg m^–^^2^)		23.3 ± 5.3	23.7 ± 2.5	24.5 ± 0.7	18.0 ± 0.0	23.2 ± 4.1
FEV1 (litres)		1.6 ± 1.1	1.9 ± 0.8	4.2 ± 0.5	3.3 ± 0.0	2.3 ± 1.3
FEV1 (% pred.)		56.8 ± 29.5	63.0 ± 24.3	97.0 ± 12.7	91.0 ± 0.0	67.9 ± 28.2
FVC (litres)		3.0 ± 1.4	3.4 ± 1.2	6.2 ± 0.3	4.7 ± 0.0	3.8 ± 1.6
FVC (% pred.)		92.3 ± 30.4	91.7 ± 12.4	113.5 ± 6.4	112.0 ± 0.0	97.3 ± 23.3
FEV1/FVC (%)		0.5 ± 0.2	0.6 ± 0.1	0.7 ± 0.1	0.7 ± 0.0	0.6 ± 02
TLC (litres)		5.0 ± 0.9	5.5 ± 1.5	8.8 ± 0.1	6.3 ± 0.0	6.0 ± 1.7
TLC (% pred.)		94.0 ± 13.3	98.0 ± 6.6	107.0 ± 2.8	104.0 ± 0.0	98.4 ± 10.4
RV (litres)		1.7 ± 0.3	2.0 ± 0.6	2.5 ± 0.1	1.7 ± 0.0	3.6 ± 5.7
RV (% pred.)		95.4 ± 8.7	115.3 ± 35.6	107.5 ± 0.7	99.0 ± 0.0	103.4 ± 19.1
DLCO (% pred.)		56.6 ± 10.4	54.0 ± 5.6	97.5 ± 7.8	77. ± 0.0	65.2 ± 18.8
KCO (% pred.)		62.2 ± 5.4	56.7 ± 4.9	92.0 ± 7.1	76.0 ± 0.0	67.4 ± 14.1
Smoking (%)	Never	5 (83.3)	0 (0.0)	2 (100.0)	0 (0.0)	7 (58.3)
	Former	0 (0.0)	2 (66.7)	0 (0.0)	0 (0.0)	2 (16.7)
	Present	1 (16.7)	1 (33.3)	0 (0.0)	1 (100.0)	3 (25.0)
Disease duration^#^		84.7 ± 49.5	43.3 ± 62.0	4 ± 0.0	33 ± 0.0	61.3 ± 49.2

Abbreviations: M = male. F = female. SD = standard deviation. BMI = body mass index. FEV1 = forced expiratory volume in 1 sec. FVC = forced vital capacity. TLC = total lung capacity. RV = residual volume. DLCO = diffusion capacity of the lung for carbon monoxide. KCO = carbon monoxide coefficient (diffusion constant).

* Continuous data are expressed as mean ± standard deviation (SD); categorical data as number and percentage (%).

^#^ Disease duration in months from time of diagnosis to study date.

### LUS findings

Multiple B-lines equalling a number of ≥3 per zone were found in three zones (2L, 3L, and 3R), corresponding to three patients, but with such focal and multiple B-lines in only one zone (Table 3). IS was not present in any of the included patients.

**Table 3.** LUS findings categorised on zones according to number and proportion.

Zone	1L	2L	3L	4L	5L	6L	7L	1R	2R	3R	4R	5R	6R	7R
	N	N	N	N	N	N	N	N	N	N	N	N	N	N
	(%)	(%)	(%)	(%)	(%)	(%)	(%)	(%)	(%)	(%)	(%)	(%)	(%)	(%)
≥3 B-lines	0 (0.0)	1 (8.3)	1 (8.3)	0 (0.0)	0 (0.0)	0 (0.0)	0 (0.0)	0 (0.0)	0 (0.0)	1 (8.3)	0 (0.0)	0 (0.0)	0 (0.0)	0 (0.0)
LS	11 (91.7)	11 (91.7)	12 (100.0)	11 (91.7)	11 (91.7)	11 (91.7)	12 (100.0)	12 (100.0)	12 (100.0)	12 (100.0)	12 (100.0)	12 (100.0)	12 (100.0)	12 (100.0)
Pl-th	0 (0.0%)	0 (0.0%)	1 (8.3%)	0 (0.0%)	0 (0.0%)	0 (0.0%)	0 (0.0%)	0 (0.0%)	0 (0.0%)	2 (16.7%)	0 (0.0%)	0 (0.0%)	0 (0.0%)	0 (0.0%)
Consolidation	0 (0.0%)	0 (0.0%)	1 (8.3%)	0 (0.0%)	0 (0.0%)	0 (0.0%)	0 (0.0%)	0 (0.0%)	0 (0.0%)	2 (16.7%)	0 (0.0%)	1 (8.3%)	0 (0.0%)	0 (0.0%)

Abbreviations: LS = lung sliding. LP = lung pulse. ≥ 3 B-lines = three or more B-lines per zone. Pl-th = pleura thickness.

Lung sliding was present in every zone on the right hemithorax among all 12 patients, but in general with a decreased presence of 91.7% on the left hemithorax.

In three patients pleural thickening was found in one zone, and observed only in the inferior lateral zones (1 patient in zone 3L with LAM, and 2 patients in zone 3R with LAM and PLCH respectively).

Lung consolidation was observed in two patients. One had sonomorphologic characteristics of bilateral pneumonia (zone 3L and 3R), and the other had uncharacteristic findings not fulfilling sonomorphologic characteristics, i.e. pneumonia, atelectasis, tumour, or pulmonary embolism.[1]

### HRCT findings

**Figure 1.**
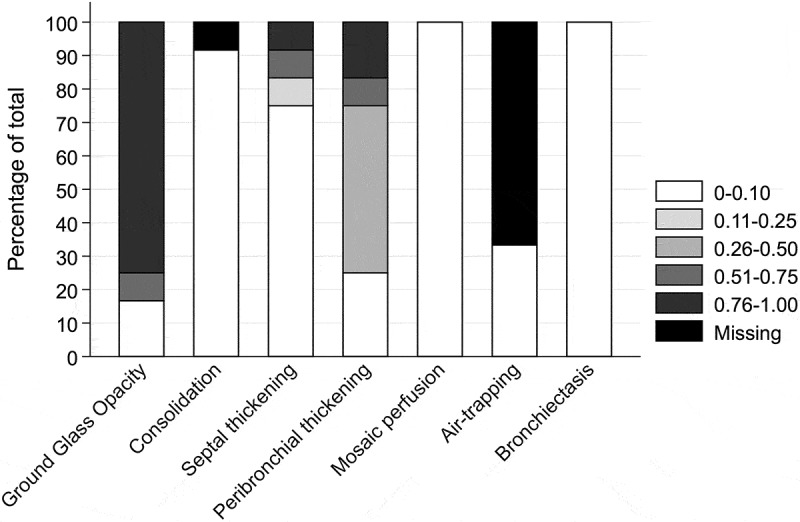
HRCT specific findings categorised according to Belmaati [26] in pentiles in numbers and proportions in percentage of total.

HRCTs prior to individual examination date were performed during 2005–2015 with a mean age of 43.8 (SD ± 43.0) months. In Figure 1 the mean Belmaati scores are presented as proportions of the total for specific HRCT findings according to the cystic lung diseases. HRCT findings consistent with consolidation, mosaic perfusion, bronchiectasis and septal thickening all showed low scores coherent with no or almost no observation of these findings. Air trapping was not found in one third of the patients; however, as expiration imaging had not been performed in eight (66.7%) of the patients’ HRCTs, air trapping as a specific finding could not be interpreted further. Half of the patients had medium scores for peribronchial thickening, almost equally distribution between low and high scores. High mean scores for GGO were present in nine patients (75%) and in only two of the patients’ HRCTs (16.7%) were no GGOs observed.

## Discussion

**Figure 2.**
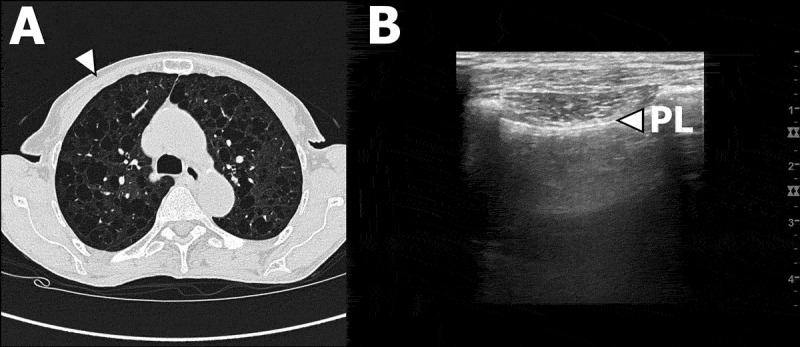
Patient #5 with LAM. (A) Transverse high-resolution computed tomography (HRCT) image of right and left upper and central lobe areas according to Belmaati.[26] White arrow corresponds anatomically to LUS zone 1 in B. (B) LUS clip of right LUS zone 1. White arrow indicates the pleural line.

**Figure 3.**
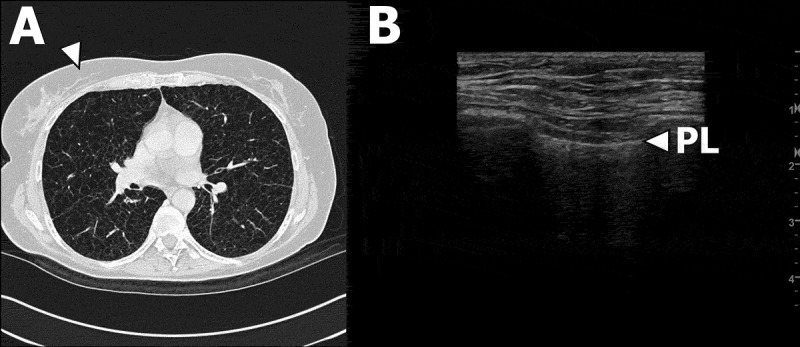
Patient #8 with LAM. (A) Transverse high-resolution computed tomography (HRCT) image of right upper and middle central and peripheral lobe areas and left upper central and peripheral lobe areas according to Belmaati.[26] White arrow corresponds anatomically to right LUS zone 2 in B. (B) LUS clip of right LUS zone 2. White arrow indicates the pleural line.

This is the first observational study to investigate the applicability and diagnostic appropriateness of LUS in a population of patients with cystic lung diseases. The key findings are that IS and pleural thickening, which are characteristic LUS findings in other subtypes of DPLD,[4–8] either could not be found or had low likelihood of presence in these patients with rare cystic lung diseases. Despite severe cystic formation on HRCTs we found normal LUS results, consisting of normal lung sliding, normal pleural thickness and no observation of IS (Figures 2 and 3).

LAM, PLCH and BHDS are rare DPLDs. The prevalence of LAM and PLCH is approximately 2–4 cases per million,[14,27] but unknown for BHDS.[17] LAM is seen almost exclusively in women with onset around 35 years of life and occurs in a sporadic form and with association to tuberous sclerosis complex (TSC), amongst whom 30–40% have affected lungs.[28] The aetiology is unknown, but generally LAM presents by an uncontrolled proliferation of smooth muscle and epithelial like cells (LAM cells), resulting in progressive cystic destruction of the lung parenchyma. LCH is mainly seen in smokers, with an equal gender distribution in 20–40 year-olds, and is due to accumulation of bone marrow derived monocytes (Langerhans’ cells) in various tissues with both multiorgan involvement (bone, skin, pituitary and lungs) and single site involvement of the lungs.[27,29] Smoking is assumed to activate and recruit Langerhans’ cells and other inflammatory cells, causing accumulation of Langerhans’ cells in the lung parenchyma, inducing nodular inflammation which over time leads to cavitating and cystic destruction.[27,29] In reverse, smoking cessation is described to improve radiological findings compatible with PLCH.[30] BHDS is an autosomal-dominant disorder caused by mutations in the gene encoding for the tumour suppressor protein folliculin (FLCN), which, by poorly defined mechanisms, cause pulmonary cysts, renal angiolipomas, and cutaneus lesions.[17]

Seventy-five per cent of the patients had diffuse GGO appearance on their HRCTs. As GGO represents diseases in which the alveolar spaces contain inflammation, oedema, haemorrhage, or thickening of the interstitium or alveolar walls, an a priori hypothesis is that presence of IS and/or a higher number of B-lines is due to an increased lung parenchymal density. However, the HRCT-GGO findings in this study contradict the LUS-IS findings. One possible explanation for this GGO-IS disagreement is a possible time-bias, since recently performed HRCTs were not available in most patients. Still, as the one patient with the study date HRCT also had general distribution of GGO, another possible explanation of the GGO-IS disagreement could be due to ”tissue compression” in the remaining lung parenchyma in consequence of progressive evolving and expanding air filled cysts.

Overall, lung sliding was a normal finding in these patients, however, one exemption was a single patient with LAM who had previously left-sided pleurodesis performed secondary to recurring pneumothorax. Due to this procedure lung sliding was not present in all scanning zones on the left hemithorax (Table 3).

Lung consolidation can be found in some types of fibrotic DPLDs as a result of fibrotic atelectasis and shrinkage, but in this study one patient showed signs of consolidation compatible with an ongoing pneumonia and one had an uncharacteristic finding, which did not add further diagnostic value. The patient in whom LUS identified pneumonic consolidation had clinical signs compatible with pneumonia (fever, dyspnoea, sputum production) and a subsequent chest X-ray revealed a pneumonic infiltrate, which was absent on previous chest X-rays.

### Strength and limitations

Whether our results are representative cannot yet be fully resolved since LUS has not been studied in cystic lung diseases, and thus we have no direct frame of reference to our results. Conversely, due to consensus on DPLD management the patients were diagnosed and treated in a tertiary specialist centre with access to MDD conferences,[21,31] so there is good reason to believe that the patients included actually suffered from rare cystic lung diseases. The single centre set-up and small cohort implies that the results cannot necessarily be applied to other tertiary specialist centres. However, based on the size of the centre from which our study cohort is included and available epidemiological data on these diseases,[14,17,27–29] a prevalence estimation of around seven cases for each of the three cystic lung diseases in focus was expected to be associated with this tertiary specialist centre. Therefore, we find our sample rather representative, which is furthermore supported by the consistent baseline findings concerning gender and age distribution and disease duration compared to existing evidence (Table 1 and 2).[14,17,27,29]

We made no distinction between the subtypes of cystic lung diseases in advance of LUS, so assessing the results as one homogeneous disease category might be a challenge. However, due to the low power we were not able to analyse potential associations between specific LUS findings and e.g. disease entity, disease distribution, age, lung physiology, and genetic disposition.

As only one of the 12 patients had a HRCT performed on the study date while the remaining 11 patients had their HRCTs performed during 2005–2015 a substantial time-bias in comparing LUS with HRCT findings might be present. In some patients the HRCT was a part of the initial diagnostics in which a previous and more active disease state including inflammation could very likely have occurred with manifestation of GGO on HRCT, and thus be the cause of the observed discrepancy between LUS-IS and HRCT-GGO. However, the chronic nature of these cystic lung diseases reduces the impact of time bias, and it is unlikely that the results of the study would have changed even if more recent HRCT examinations were available.

## Conclusion

The diagnostic value of LUS in patients with rare cystic lung diseases such as LAM, PLCH, and BHDS seems limited as normal LUS findings did not rule out severe cystic lung disease. In a clinical setting such knowledge, however, is important to be aware of since LUS is not recommended as the first choice of radiological imaging when aiming to diagnose these diseases. Nevertheless, LUS may still be clinically useful, since abnormal LUS findings should alert the clinician of possible complications or concomitant disease.
